# RAGE signaling regulates the progression of diabetic complications

**DOI:** 10.3389/fphar.2023.1128872

**Published:** 2023-03-16

**Authors:** Kensei Taguchi, Kei Fukami

**Affiliations:** Department of Medicine, Division of Nephrology, Kurume University School of Medicine, Kurume, Fukuoka, Japan

**Keywords:** receptor for advanced glycation endproducts, AGEs, DNA aptamer, diabetic nephropathy, chronic kidney disease

## Abstract

Diabetes, the ninth leading cause of death globally, is expected to affect 642 million people by 2040. With the advancement of an aging society, the number of patients with diabetes having multiple underlying diseases, such as hypertension, obesity, and chronic inflammation, is increasing. Thus, the concept of diabetic kidney disease (DKD) has been accepted worldwide, and comprehensive treatment of patients with diabetes is required. Receptor for advanced glycation endproducts (RAGE), a multiligand receptor, belonging to the immunoglobulin superfamily is extensively expressed throughout the body. Various types of ligands, including advanced glycation endproducts (AGEs), high mobility group box 1, S100/calgranulins, and nucleic acids, bind to RAGE, and then induces signal transduction to amplify the inflammatory response and promote migration, invasion, and proliferation of cells. Furthermore, the expression level of RAGE is upregulated in patients with diabetes, hypertension, obesity, and chronic inflammation, suggesting that activation of RAGE is a common denominator in the context of DKD. Considering that ligand–and RAGE–targeting compounds have been developed, RAGE and its ligands can be potent therapeutic targets for inhibiting the progression of DKD and its complications. Here, we aimed to review recent literature on various signaling pathways mediated by RAGE in the pathogenesis of diabetic complications. Our findings highlight the possibility of using RAGE–or ligand–targeted therapy for treating DKD and its complications.

## 1 Introduction

Diabetes mellitus (DM) is emerging as a major public health concern worldwide. One–seventh of the adults in the United States are diagnosed with diabetes, and the prevalence increases to 29.2% among those aged 65 years or older (Hamman et al., 2014; Divers et al., 2020). The Epidemiology of Diabetes Interventions and Complications study, an observational follow–up study to the Diabetes Control and Complications Trial, revealed that poor initial glycemic control is linked to a high prevalence of future diabetic complications, even if the recent glycemic control was intensive (Lachin et al., 2015). This phenomenon has been named “metabolic memory.” Strictly controlling the glucose level only during the first year after the diagnosis of diabetes is considerably associated with the future risk of diabetic complications and mortality, even after adjusting for glycemic control in the second year after diagnosis (Laiteerapong et al., 2019). Furthermore, hyperglycemia–derived substances that accumulate abnormally in organs may cause diabetic complications. High glucose level stimulates the formation of advanced glycation endproducts (AGEs) that get deposited and crosslink between long turnover proteins, such as collagens, which are not easily degraded, thereby causing long–lasting organ dysfunction independent of the current glucose levels. Thus, AGEs are believed to contribute to “metabolic memory” under diabetic conditions.

An estimated one–third of the patients with diabetes develop kidney injury during their lifetime. Notably, the incidence of cardiovascular events and cardiac death notably increases with the worsening of renal function (Jankowski et al., 2021). It has been suggested that excessive accumulation of AGEs due to the inability to excrete is involved in the development of endothelial dysfunction, leading to atherosclerosis and cardiovascular events (Su et al., 2008). As the incidence of cardiovascular events is positively correlated with the serum concentration of AGEs (Kerkeni et al., 2013), AGEs have been proposed as the major component in the formation of a vicious cycle between the heart and kidney in patients with diabetes. Direct deposition of AGEs in the tissue induces organ damage, but AGEs also act cytotoxically, activating intracellular signaling by interacting with the receptor for AGEs (RAGE) in various cell types. In fact, the upregulation of RAGE expression is associated with endothelial cell damage *via* its interaction with AGEs (Bucciarelli et al., 2002). Additionally, RAGE expressed on phagocytes promotes the transformation to foam cells and accelerates the infiltration of foam cells into atherosclerotic lesions that are associated with vascular complications in patients with diabetes (Daffu et al., 2015).

RAGE is a multiligand receptor belonging to the immunoglobulin superfamily. Various types of ligands bind to RAGE, leading to pathological inflammation, activation of the renin–angiotensin–aldosterone system (Fukami et al., 2004; Yokoyama et al., 2021), initiation of TGF–β signaling (Brizzi et al., 2004), induction of aberrant angiogenesis (Chen et al., 2014) and adhesion signaling (Sessa et al., 2014). Thus, RAGE is involved in the development of various renal diseases, including obesity–related nephropathy, hypertensive kidney injury, and diabetic kidney injury. Owing to the recent improvements in diabetes and hypertension therapy, the typical clinical course of kidney injury in patients with diabetes has changed, and the concept of diabetic kidney disease (DKD) has been globally accepted. DKD can be induced by several complicated factors, such as diabetes, hypertension, obesity, and hyperlipidemia, implicating that upregulation of RAGE expression seems to be a common denominator among these diseases; thus, RAGE is an optimal target for preventing DKD and its complications. Here, we aimed to review various signaling pathways mediated by RAGE in the pathogenesis of diabetic complications and highlight the possibility of using a RAGE–targeted strategy for inhibiting diabetes–induced longitudinal organ damage.

## 2 *In vivo* formation of AGE

Louis Camilli Maillard (1878–1936) discovered that heating a mixture of glycine and glucose produces brown substances, and the reaction was later named the Maillard reaction. The carbonyl base of reducing sugars, such as glucose, reacts non-enzymatically with the amino base at the N–terminus or lysine residues of amino acids to form a Schiff base. After relatively stable Amadori compounds are formed, intermediate metabolites, namely, “dicarbonyls,” are produced *via* various reactions, such as dehydration–condensation, oxidation, reduction, and intermolecular crosslink formation. Dicarbonyls react further to form irreversible products, the so–called AGEs. In addition to Maillard reactions, the polyol pathway, glycolysis, lipid peroxidation, and glucose autoxidation are involved in the formation of reactive dicarbonyls, such as methylglyoxal, glyoxal, and 3–deoxyglucosone. As proteins with slow metabolic turnover (e.g., collagen fibers) are exposed to reducing sugars in the long term, lysine and arginine residues of collagens are likely to be modified by the Maillard reaction to form crosslinks. Thus, the degradation of AGEs is presumably delayed compared to that of normal proteins because of the polymerization of proteins, reduced solubility, and impaired reactivity to proteases. AGEs are a general term for a group of compounds produced by glycation. However, glycation is required for the production of pyralin and crosslin; in contrast, Nε–carboxymethyllysine (Nε–CML) and pentosidine are formed *via* oxidation. It is generally accepted that negatively charged or oligomerized proteins are more likely to interact with RAGE and its ligands than the other proteins. Modification of lysine and arginine during AGE formation renders the protein surface negatively charged, which enables binding to RAGE and amplifies RAGE–mediated inflammation and adhesion. Recently, Nafty et al. demonstrated that glycation alters the helical conformation of serum albumin and promotes the formation of β–sheets enriched with amyloid fibrils (Naftaly et al., 2021). Serum albumin becomes insoluble and aggregates during co–incubation with methylglyoxal, glycolaldehyde (Naftaly et al., 2021) and ribose–modified albumin, thereby forming amyloid–like aggregates that induce cytotoxicity (Wei et al., 2009), which is linked to the development of Alzheimer’s disease (Tu et al., 2016). There are a wide variety of pathways involved in the formation of AGEs and the other RAGE ligands.

## 3 Therapeutic strategies aimed at inhibition of AGEs formation in diabetic complications

The metabolic excretion pathway of AGEs is primarily through the kidneys. Approximately 50%–80% of Exogenous AGEs is excreted by kidney; but AGEs remain deposited in the kidney (Miyata et al., 1998; Liang et al., 2020). In diabetes, hyperglycemia increases AGE production, leading to excessive deposition of AGEs in the kidney and various kidney cells, including the glomerular basement membrane, mesangial cells, podocytes, tubular cells, and vascular endothelial cells, thereby promoting cellular damage. Aminoguanidine, a guanidine derivative, traps reactive dicarbonyls and prevents AGE formation. Administration of aminoguanidine to streptozotocin (STZ)–induced diabetic mice decreased urinary albumin excretion and improved diabetic glomerulosclerosis (Soulis–Liparota et al., 1991). In a clinical trial (ACTION I) including diabetic patients with massive proteinuria, aminoguanidine slowed the decline in estimated glomerular filtration rate (eGFR) and prevented retinopathy, as assessed using the Early Treatment of Diabetic Retinopathy Study Score (Freedman et al., 1999; Kern and Engerman, 2001). Another water–soluble vitamin B6 compound, pyridoxamine, similarly inhibited the formation of AGEs by scavenging reactive dicarbonyls, free radicals, and metal chelate inhibition (Booth et al., 1996). The administration of pyridoxamine mixed in drinking water to STZ–induced diabetic rats decreased the urinary albumin excretion rate, kidney weight, and plasma creatinine concentration, with a decrease in cutaneous CML and time required for protease digestion of tissue proteins (Xu et al., 2005). Thus, the inhibition of AGE formation can inhibit the onset and progression of diabetic nephropathy.

Another class of compounds, known as crosslink breakers, targeted against the AGE–RAGE axis can break the cross–linking between AGEs and extracellular matrix, such as collagens. Alagebrium (ALT–711), N–phenacyl thiazolium, and pyridinium bromide derivatives are crosslink breakers. Notably, alagebrium does not affect the modification of natural carbohydrates to proteins, intramolecular crosslink, and peptide bonds responsible for maintaining the normal integrity of the collagen chain. Thus, the normal structure and function are preserved, while abnormal crosslink is reduced. In *in vitro* experiments, the digestion of diabetic rats’ tail using pepsin was reduced owing to AGE–regulated crosslink, which was significantly improved by co–incubation with ALT–711 (Wolffenbuttel et al., 1998). ALT–711 was found to reduce the accumulation of pentosidine and attenuate kidney injury in db/db diabetic mice by decreasing NADPH oxidase activation (Park et al., 2011). Watoson et al. demonstrated that treatment with ALT–711 reduced glomerular matrix accumulation and inhibited the gene expression of inflammation markers, including monocyte chemotactic protein–1 (MCP–1), ICAM–1, and CD11b, even when RAGE was genetically deleted in STZ–induced diabetic Apo–E knockout mice (Watson et al., 2012). Considering that ALT–711 targets cross–linked AGEs, the protective effect of ALT–711 is partially RAGE–independent. Clinical trials have demonstrated that not only kidney injury but also atherosclerosis seems to be attenuated by the treatment with ALT–711. ALT–711 (210 mg) was administered once daily to patients with resting arterial pulse pressures >60 mmHg and systolic pressures >140 mmHg for 56 days, and preexisting antihypertensive medications were continued. It has been demonstrated that ALT–711 improves total arterial compliance and reduces pulse wave velocity better than the placebo (Kass et al., 2001). In 23 patients with diastolic heart failure, ALT–711 reduced the left ventricular mass, attenuated the left ventricular diastolic filling, and improved the quality of life. However, ejection fraction, blood pressure, and peak exercise oxygen consumption were not affected by the administration of ALT–711 (Little et al., 2005). In addition, exercise tolerance was not improved by the administration of ALT–711 in 102 patients with systolic heart failure having ejection fraction ≤0.45 (Hartog et al., 2011).

While the protective effects of the agents directed against AGE have been revealed in animal experiments and clinical trials as described above, some clinical trials have stopped due to their severe adverse effects including flu–like symptoms, liver dysfunction, vasculitis, and formation of antinuclear antibody (Freedman et al., 1999). Thus, clinical application of all the agents directed against AGEs has been abandoned. In response to this trend, RAGE, a main receptor for AGEs, has recently drawn attention as a therapeutic target for inhibiting AGEs–RAGE axis.

## 4 Characteristics of structure and regulation of RAGE

RAGE, a single transmembrane receptor, belongs to the immunoglobulin superfamily (Yonekura et al., 2003) and, in bovine lungs, has been identified as a receptor that binds to AGEs (Neeper et al., 1992). RAGE, a 44–55 kDa protein, is composed of an extracellular region, including one V–domain and two C–domains to which many ligands bind, a single transmembrane helix, and a cytoplasmic tail, which is required for RAGE–mediated intracellular signaling. RAGE is a pattern recognition receptor; thus, AGEs (Huttunen et al., 1999), high–mortality group box–1 (HMGB–1) (Hori et al., 1995), S100/calcineurins (Hofmann et al., 1999), amyloid fibrils β (Chaney et al., 2005), lipopolysaccharides (LPS) (Qin et al., 2014), and segmented DNA and RNA (Sirois et al., 2013) can bind to the extracellular domain of RAGE. The binding of the ligands to RAGE provides stability for the formation of oligomers and facilitates cell signaling that regulates the migration, proliferation, and adhesion of several cell types (Yatime and Andersen, 2013). The C1 domain of RAGE has a positively charged patch, whereas the C2 domain is negatively charged. As AGEs and acidic S100 proteins are negatively charged, they are electrically attracted to the C1 domain. Furthermore, these ligands were shown to bind to RAGE in a competitive manner (Liu et al., 2008). RAGE also recognizes phosphatidylserine, which enables phagocytes to consume apoptotic cells. However, the coexistence of HMGB–1 and phosphatidylserine competitively inhibit the interaction between phosphatidylserine and RAGE, thereby blocking the phagocytosis of apoptotic cells (Liu et al., 2008). Moreover, Yamamoto et al. demonstrated that oxytocin is another candidate RAGE ligand and that oxytocin is transported into the brain *via* its interaction with RAGE on the brain capillary endothelial cells, which is important for regulating behavioral actions, including parenthood and social connection (Yamamoto et al., 2019).

A positive feedback mechanism regulates RAGE expression. When ligands bind, the activated RAGE increases nuclear factor–kappa B (NF–κB) expression, which promotes the upregulation of RAGE expression *via* transcriptional activity. This response leads to the further activation of NF–κB, which strongly amplifies RAGE–mediated cell signaling. In contrast, RAGE has an autoregulatory system. For instance, after HMGB–1 binds to RAGE, the extracellular domain of RAGE is cleaved by disintegrin and metalloproteinase domain–containing protein 10 (ADAM10) and matrix metalloproteinase (MMP) 9 in a phosphatidylinositol–3 kinase (PI3K)– or protein kinase C (PKC)–dependent manner. Ectodomain RAGE, known as soluble RAGE (sRAGE), is released by proteolysis and probably infiltrates the bloodstream and act as a decoy receptor by neutralizing circulating ligands. sRAGE can not only indicate the expression level of full–length RAGE expressed on the cell membrane but also can predict the development of diabetes (Thomas et al., 2011), cardiovascular disease (Thomas et al., 2011; Tang et al., 2017), and various inflammatory diseases (Manganelli et al., 2019). In particular, a decrease in serum sRAGE level due to a decrease in ADAM10 levels under diabetic conditions may predispose patients with type 2 diabetes to acute coronary syndrome (Ragavi et al., 2022). The shedding of the ectodomain RAGE is believed to abrogate ligand–mediated intracellular signaling, inducing negative feedback. However, Braley et al. recently demonstrated that resistance to ectodomain RAGE shedding inhibits RAGE ligand–dependent cell migration and spreading by suppressing phosphorylation of Src, ERK, AKT, and p38 (Braley et al., 2016). Thus, the proteolysis of RAGE might affect RAGE–mediated cell signaling and cellular functions, which can be a novel therapeutic strategy for regulating the pathological role of RAGE.

## 5 Intracellular signaling of RAGE activated by engagement of the ligands

**FIGURE 1:**
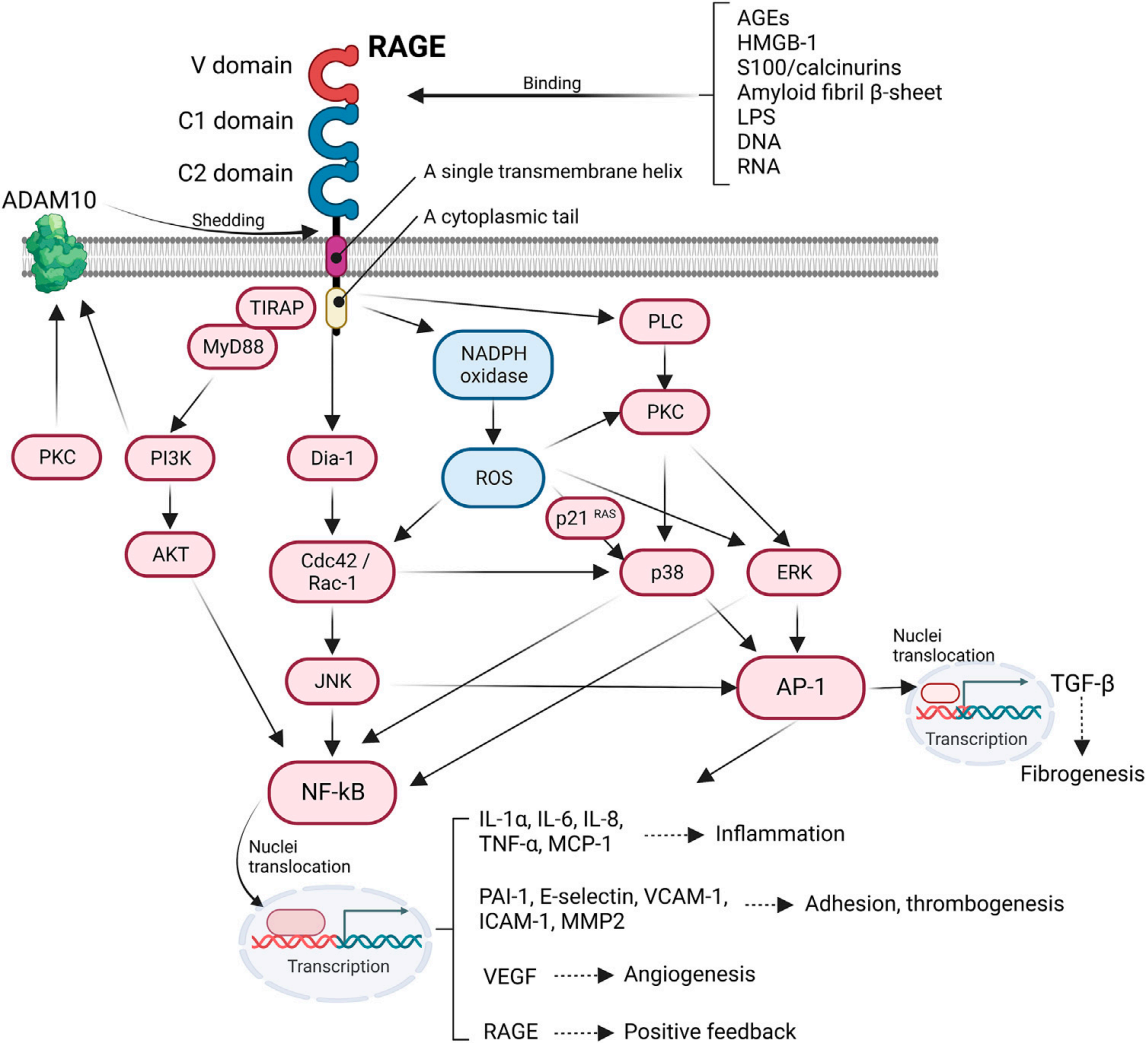
Intracellular RAGE signaling. The engagement of RAGE with several ligands including AGEs, HMGB-1, S100/calcinurins is involved in profibrotic and inflammatory response, cell adhesion, angiogenesis, and thrombogenesis.

The engagement of the ligands with RAGE activates several intracellular signaling pathways that regulate a wide range of cellular functions, such as migration and cytoskeleton organization. In addition, RAGE activation has a considerable effect on transcriptional profiles, including pro–inflammatory cytokine, adhesion–related, and profibrotic genes. After interacting with ligands, the cytoplasmic domain of RAGE interacts with the formin homology (FH1) domain of Dia–1, which then activates Rho GTPases Rac–1 and Cdc42, which are essential for transducing signals linking plasma membrane receptors to the organization of the cytoskeleton and regulating gene transcription (Hudson et al., 2008). Rac–1 or Cdc42 induces the activation of JNK and p38 mitogen–activated protein kinase (MAPK) (Philips et al., 2000), which in turn activates the pro–inflammatory master transcription factor NF–κB (Tóbon–Velasco et al., 2014) and activator protein 1 (AP–1) (Lee et al., 2021). The activated NFκB translocate into the nuclei and promote the expression of various pro–inflammatory genes encoding cytokines and chemokines (e.g., IL–1α, IL–6, and TNF–α), cellular adhesion factors (vascular cell adhesion molecule–1; VCAM–1), intracellular cell adhesion molecule–1 (ICAM–1), and E–selectin. Additionally, NF–κB is associated with inflammasome regulation. Moreover, RAGE–induced activation of NF–κB and AP–1 accelerates the transcriptional production of TGF–β, thereby causing extracellular matrix production and fibrosis (Wu et al., 2018). RAGE recruits two adaptor proteins, MyD88 and TIRAP, in a PKC–dependent manner to form multiprotein complexes that are required to transduce signals to the downstream target (Allen et al., 2022). Thus, the ligands binding to RAGE activate AKT *via* TIRAP/MyD88 (Sakaguchi et al., 2011), which, in turn, perpetuates the activation of NF–κB, thereby amplifying the pro–inflammatory response (Figure 1).

Reactive oxygen species (ROS) serve as secondary messengers in intracellular signal transduction in a variety of cellular processes. However, ROS probably induces cellular injury through protein oxidation and nitrosylation, impairing enzymatic processes (Damgaard et al., 2017), growth factors (Stadtman and Levine, 2000), and lipid oxidation (Hogg et al., 1993) and by introducing double–strand breaks by nucleic acid oxidation, thus initiating maladaptive necrosis and apoptosis (Auten et al., 2002). Cellular ROS production is mainly induced by nicotinamide adenine dinucleotide phosphate (NADPH) oxidase. NADPH oxidase is composed of two membrane–bound subunits (p22phox and gp91phox), three cytosolic subunits (p67phox, p47phox, and p40phox), and a small G–protein that catalyzes the generation of superoxide anion radicals and subsequent hydroxyl radicals from molecular oxygen (Sohn et al., 2000). Ligation of RAGE increases the expression of NADPH oxidase subunits and activates NADPH oxidase, contributing to the formation of ROS (Kass et al., 2001). RAGE–induced ROS stimulates the expression of p21 RAS (Lander et al., 1997), which can also activate NF–κB and AP–1, promoting an inflammatory response *via* the MAPK/extracellular signal–regulated kinase (ERK) pathway (Zhou et al., 2003) (Figure 1). RAGE signaling is important for inducing aberrant inflammatory responses and oxidative stress–induced cellular damage, which is known to be involved in the pathogenesis of Alzheimer’s disease (Liu et al., 2008), rheumatoid arthritis (de Groot et al., 2011), and inflammatory bowel disease (Body-Malapel et al., 2019).

Wendt et al. found that levels of RAGE ligands other than AGEs, such as S100/calgranulins, are also increased in diabetic nephropathy, and these are considerably involved in inflammatory cell infiltration (Wendt et al., 2003). Similarly, serum HMGB1 level was significantly increased in patients with type 2 diabetes compared to that in healthy individuals, which was considerably positively correlated with concentrations of inflammatory cytokine, such as TNF–α and IL–6 (Chen et al., 2015). These findings suggest that the regulation of ligand–RAGE interactions is required for the pathogenesis of diabetic nephropathy. Moreover, the induction of type 1 diabetes in C57BL/6J mice with STZ results in urinary albumin excretion and renal dysfunction, as well as renal enlargement, increased glomerular mesangial matrix, progressive glomerulosclerosis, and upregulation of TGF–β and vascular endothelial growth factor (VEGF) expression. However, these changes were significantly inhibited in RAGE knockout mice (Reiniger et al., 2010). In addition, administration of RAGE–neutralizing antibodies to db/db mice reduced urinary albumin excretion, enlargement of mesangial areas, and thickening of the basement membrane, characteristic of early diabetic nephropathy (Jensen et al., 2006). Reinger et al. showed that crossing OVE26 mice, a spontaneous type 1 diabetic mouse, with RAGE knockouts improved glomerulosclerosis and led to the thickening of the glomerular basement membrane with reduced podocyte foot process effacement and preserved renal function. Thus, RAGE is one of the main contributors to the progression of diabetic nephropathy, and the inhibition of RAGE may be beneficial for preventing kidney injury in patients with diabetes.

## 6 The engagement of RAGE with ligands promotes vascular complications

AGEs enhance ROS production in vascular endothelial cells and subsequently induce redox–sensitive cytokines associated with atherosclerosis, including MCP–1, MMP–9, and plasminogen activator inhibitor–1 (PAI–1), thus leading to atherosclerotic plaque formation and instability in the coronary arteries (Bucala et al., 1991; Goldin et al., 2006). AGEs increase vascular permeability, promote migration of macrophages and T cells to the arterial intima, and decrease vascular relaxation by inhibiting the production of endothelial nitric oxide synthase (eNOS) (Soro–Paavonen et al., 2008). RAGE expression level is upregulated in atherosclerotic plaques of patients with diabetes and is co–expressed with inflammatory markers, such as cyclooxygenase–2 and MMPs. In addition, RAGE expression upregulation is observed primarily in macrophages residing in unstable plaques. STZ–induced diabetic Apo–E knockout mice exhibited prominent atherosclerotic lesions, whereas genetic inhibition of RAGE expression reduced the area of atherosclerosis without affecting glucose or fat metabolism in diabetic Apo–E knockouts (Tikellis et al., 2008). Further, it has been reported that RAGE knockouts show a suppressive effect on the development of atherosclerosis in non-diabetic individuals fed with high–fat diets (Brown et al., 2007). These findings suggest that the AGE–RAGE system is involved in vascular complications *via* the activation of immune cells, inflammation, and endothelial cell dysfunction in diabetes.

**FIGURE 2:**
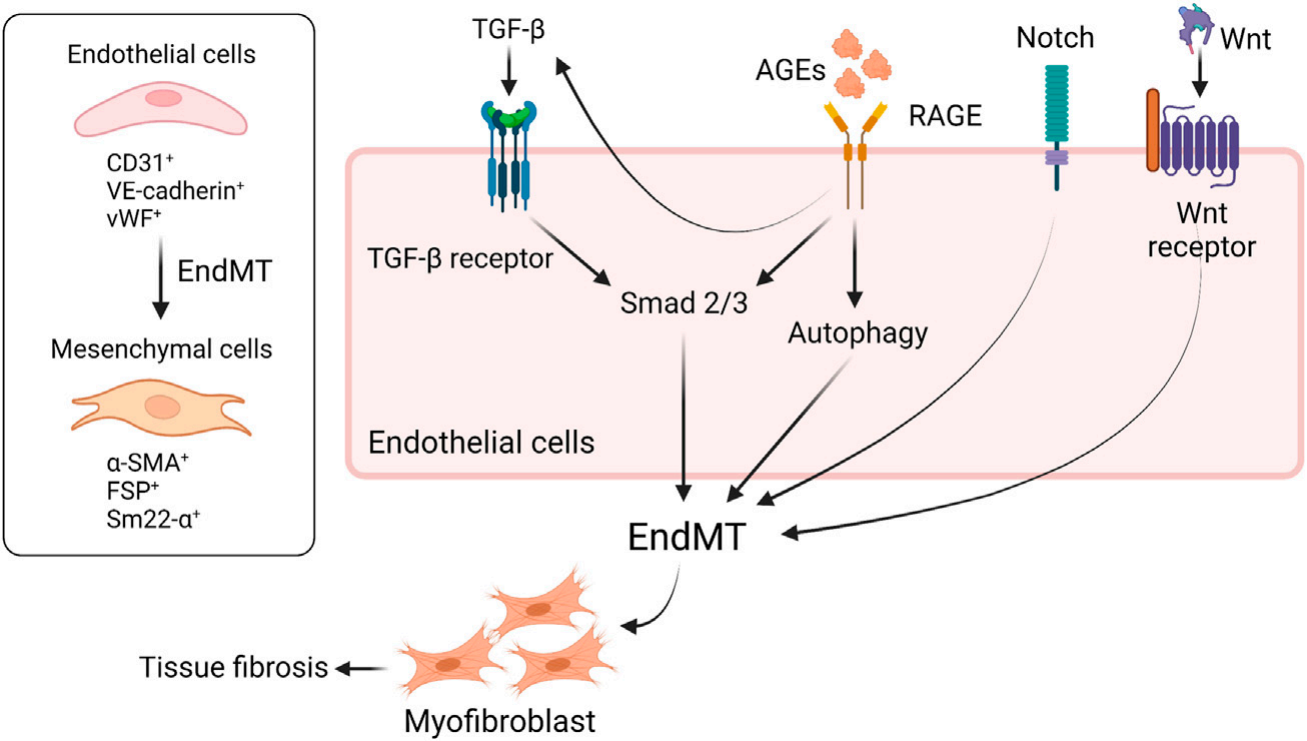
RAGE and other signaling are implicated in EndMT in diabetes. EndMT contributes to tissue fibrosis in diabetic condition. The engagement of RAGE with AGEs induces EndMT through the activation of Smad2/3 or aberrant upregulation of autophagy. TGF-β also activates Smad2/3, leading to EndMT. RAGE-induced increase in TGF-β amplifies EndMT, resulting in tissue fibrosis. Besides, Notch signaling or Wnt/β-catenin pathway is involved in EndMT as well. EndMT, endothelial-to-mesenchymal transition; TGF-β, tissue growth factor-b; RAGE, receptor for advanced glycation endproducts; VE-cadherin, vascular endothelial-cadherin; vWF, von Willebrand factor; α-SMA, a-smooth muscle actin; FSP, fibroblast-specific protein; sm22-α, smooth muscle protein 22.

Endothelial–mesenchymal–transition (EndMT) contributes to tissue fibrosis in diabetic condition. Several pathways including TGF–β (Cooley et al., 2014), Notch pathway (Wang et al., 2018), Wnt/β–catenin signaling pathway (Li et al., 2019), and AGEs–RAGE axis are involved in EndMT (Figure 2). Lineage tracing analysis of endothelial cells using genetic engineering techniques showed that some population of adult bovine endothelial cells differentiates into smooth muscle cells (Zeisberg et al., 2007; LeBleu et al., 2013). Li et al. demonstrated that 30%–50% of fibroblast in STZ–induced diabetic kidneys were endothelial–origin (Li et al., 2009), which was driven by TGF–β and subsequent Smad3 activation (Li et al., 2010). AGEs are also shown to promote EndMT *via* activation of Smad3 through the interaction with RAGE (Li et al., 2010). As an alternative pathway, AGE induces TGF–β production *via* downregulation of SIRT1 in endothelial cells and then increased TGF–β accelerates EndMT (He et al., 2015). Also, engagement of RAGE with AGEs on endothelial cells accelerates aberrant autophagy, promoting EndMT and tissue fibrosis in rodents; thus, inhibition of RAGE is thought to reduce fibrosis through suppressing EndMT (Zhang et al., 2021). In addition, glucocorticoid receptor (GR), a nuclear hormone receptor expressed ubiquitously in most cell types, is known to regulate EndMT in diabetes. Srivastava et al. clearly demonstrated that loss of GR in endothelial cells promotes EndMT *via* activation of WNT signaling, leading to reprogramming of cytokines, resulting in renal fibrosis in diabetic rodents (Srivastava et al., 2021a). Similarly, podocyte GR is essential for maintaining endothelial homeostasis. In fact, podocyte–specific deletion of GR upregulates WNT signaling pathway and suppresses fatty acid oxidation, leading to disruption of endothelial homeostasis and profibrotic process. The profibrotic phenotype in podocyte–specific loss of GR might contribute to EndMT (Srivastava et al., 2021b). Meanwhile, fibroblast growth factor (FGF) signaling is known to play a central role for endothelial barrier system and endothelial survival (Murakami et al., 2008). The engagement of FGFR with FGFs inhibits TGF–β production and then suppresses EndMT (Chen et al., 2012). By contrast, endothelial deficiency of FGFR1 induces EndMT which, in turn, results in endothelial–mesenchymal transition (EMT), a different program to promote tissue fibrosis, in neighboring tubules (Li et al., 2020), suggesting that FGFR1 is a therapeutic option to prevent the progression of diabetic organ damage. A previous study showed that the protective effect of exogenous administration with FGF1 is dependent on RAGE (Zheng et al., 2021); thus, inhibition of RAGE might be able to mimic the protective phenotype of FGF1 in diabetes. SIRT3, a mitochondrial sirtuin, plays a role in mitochondrial integrity and metabolism; thereby, SIRT3 has an anti–fibrotic effect in diabetic kidneys. Interestingly, endothelium–specific loss of SIRT3 promotes EndMT with the increase in fibrosis in diabetic kidneys (Srivastava et al., 2021c). In addition to EndMT, activation of Wnt signaling is associated with tubular EMT (Matsui et al., 2007), podocyte injury (Dai et al., 2009), and mesangial cellular injury (Tung et al., 2018) in diabetic kidney disease. Not only is Hedgehog pathway essential for kidney development, but also implicated in the progression of tissue fibrosis (Kramann et al., 2015). Miyata et al. demonstrated that urine concentration of hedgehog interacting proteins (Hhip), functioning as a decoy receptor and antagonizing hedgehog signaling, is upregulated in glomerular endothelial cells in diabetic kidneys and the urine concentration is elevated earlier than albuminuria in diabetic patients. The finding suggests that urine Hhip might predict the onset of diabetic nephropathy (Miyata et al., 2020). Taken together, novel therapeutic targets as mentioned here have recently been identified and will be investigated if those including RAGE–regulated EndMT can be applied to clinical practice.

## 7 RAGE mediates endothelial permeability in diabetic conditions

Approximately half of the patients with type 2 diabetes and one–third with type 1 diabetes develop chronic kidney disease (CKD), which is clinically defined by the presence of impaired renal function, elevated urinary albumin excretion, or both (Dwyer et al., 2012). Microalbuminuria is a hallmark of early stage diabetic nephropathy and usually indicates damage to the glomerular filtration barrier due to ultrastructural changes in podocytes and glomerular endothelial cells rather than alterations in glomerular pressure or filtration rate alone. There is increasing evidence that RAGE activation compromises microvascular barrier function, leading to endothelial hyperpermeability (Xiong et al., 2011). AGEs induce intercellular gap formation and enhance permeability *via* Rho activation in a RAGE–dependent manner in human umbilical vein endothelial cells (Hirose et al., 2010). Another study demonstrated that RAGE mediates the phosphorylation of β–catenin and its nuclear translocation, which in turn promotes the transcription of ADAM10, which cleaves vascular endothelial cadherin (VE–cadherin), an endothelial–specific adhesion molecule located at junctions between endothelial cells, thereby contributing to the disruption of cell–cell adhesion (Weng et al., 2021). Although angiotensin II (Ang II) is the main contributor in the pathogenesis of diabetic nephropathy, Ang II also induces endothelial hyperpermeability. Ang II induces the release of HMGB–1 from endothelial cells, which then binds to RAGE, leading to a decrease in VE–cadherin level. Notably, the blockade of RAGE using sRAGE attenuated the Ang II–induced increase in endothelial hyperpermeability (Jeong et al., 2019). A recent study reported similar findings regarding the HMGB–1–RAGE axis enhancing barrier permeability in human pulmonary microvascular endothelial cells (Zhao et al., 2021). Sepsis–associated acute kidney injury is a common complication in hospitalized and critically ill patients. During sepsis, endothelial permeability is severely augmented, which promotes kidney injury. Koga et al. revealed that neutralization of RAGE improved the survival rate of LPS–induced septic mice with a reduction in HMGB–1 concentration and ROS production (Koga et al., 2021). The protective effect of RAGE inhibition in sepsis can be explained by the restoration of endothelial permeability. Thus, there is growing evidence that RAGE is involved in endothelial permeability, which contributes to the development of kidney injury in patients with DM, as well as other kidney diseases.

## 8 Cooperation between AGE–RAGE and asymmetric dimethyl arginine (ADMA) promotes endothelial dysfunction in diabetic conditions

Since Bucala et al. reported the effect of nitric oxide (NO) supplementation on AGEs in 1991 (Bucala et al., 1991), various studies have been performed to evaluate the relationship between AGEs and NO. In cultured vascular endothelial cells, eNOS activity was suppressed by serum isolated from patients with CKD, suggesting that AGEs, ADMA, homocysteine, and other substances that accumulate in the body owing to impaired renal function may reduce the biological activity of NO. Furthermore, in a clinical trial on 51 patients with CKD, the serum concentration of AGEs increased with the progression of CKD. Considering their inverse correlation with the post–occlusive reactive hyperemia index, an index of microvascular endothelial function (Linden et al., 2008), AGEs inactivate NO, thereby possibly contributing to atherosclerosis development and subsequent kidney injury. A possible mechanism by which AGEs suppress NO activity involves ADMA, an endogenous NO inhibitor. A strong positive correlation has been demonstrated between serum AGE and ADMA levels in patients with CKD (Nakamura et al., 2009). When treating cultured vascular endothelial cells with AGEs, AGEs enhance the expression level of protein arginine methyltransferases (PRMTs), the rate–limiting enzyme in ADMA production, resulting in an increase in intracellular ADMA production (Ojima et al., 2013). Ando et al. investigated the relationship between serum AGEs and ADMA in CKD patients with DM and identified that flow–mediated vasodilatation, an index of vascular endothelial function, decreased as serum AGE and ADMA levels increased (Ando et al., 2013). Furthermore, coincubation of cultured proximal tubules with AGEs reduced the expression level of dimethylarginine dimethylaminohydrolase (DDAH)–2, an ADMA–metabolizing enzyme, in cultured vascular endothelial cells, leading to an increased ADMA concentration in the supernatant. Taken together, accumulation of AGEs due to diabetes, progressive renal dysfunction, and aging is linked to increased ADMA concentration *via* the increase in expression of PRMTs and reduction of DDAH expression, leading to NO deficiency and vascular endothelial dysfunction in diabetic conditions.

## 9 RAGE plays a pathological role in vasculopathy in non-diabetic condition

Vascular calcification in the coronary arteries, carotid arteries, and aorta is a critical risk factor for the development of cardiovascular events in patients with CKD. Vascular calcification leads to decreased arterial compliance, increased pulse pressure, and an increased left ventricular afterload. Vascular smooth muscle cells (VSMCs) are the primary cells responsible for vascular formation. When RAGE was overexpressed using an adenoviral transduction technique in human aorta–derived VSMCs (HASMC), HMGB1 enhanced the expression of osteoblast–specific alkaline phosphatase (ALP) gene and osteoblast differentiation factors, such as msh homeobox and Runx2, in a RAGE–dependent manner. In addition, DAPT, a Notch signaling inhibitor, substantially prevented RAGE–mediated differentiation of HASMC to osteoblasts, suggesting that RAGE is involved in vascular calcification *via* the Notch pathway (Suga et al., 2011). Notably, Ang II–induced HMGB1 stimulates calcium deposition in human aortic smooth muscle cells and Apo–E KO mice, which was inhibited by neutralizing anti–HMGB–1 antibody and sRAGE (Jeong et al., 2022); the findings suggest that HMGB–1 or other ligands released from VSMCs bind to RAGE in a paracrine manner, contributing to arterial osteogenesis.

Low–density lipoprotein (LDL) also undergoes glycation and oxidation. Apolipoprotein B, a specific LDL protein, is easily glycated and modified under hyperglycemic conditions. Furthermore, the uptake of glycated LDL into the arterial intima *via* scavenger receptors, such as SR–A and CD36, promotes the formation of foam cells and progresses to atherosclerotic lesions (Hedrick et al., 2000). Modified LDL enhances RAGE–mediated uptake by macrophages and promotes the transition of macrophages to foam cells. Foamy macrophages secrete various chemokines and growth factors, which induce the migration and proliferation of smooth muscle cells, as well as the invasion of monocytes into the subendothelium. The binding of ligands to RAGE accelerates the production of adhesion factors, such as ICAM–1 and VCAM–1, in endothelial cells and induces the aggregation of monocytes and T lymphocytes into the subendothelial tissue. Inhibition of the interaction between cytoplasmic tail RAGE and DIAPH 1 blocks cell formation, cell invasion, activation of NF–κB, and upregulation of pro–inflammatory cytokine expression in microphages isolated from mice. Thus, RAGE–mediated intracellular signal transduction regulates macrophage activation, contributing to vascular inflammation at atherosclerosis sites.

In addition, the receptor for nuclear factor B (RANK)–RANK ligand (RANKL)–osteoprotegerin (OPG) pathway and the AGEs–RAGE system orchestrate the formation of vascular calcification under diabetic conditions. RANKL, a member of the TNF superfamily, binds to RANK and induces the intracellular activation of NF–κB, which in turn increases the expression of osteogenic genes *via* transcriptional activity. OPG inhibits the RANKL–RANK interaction, functioning as a decoy receptor for RANKL. In cultured rat aorta–derived muscle cells, RANKL induces deposition of inorganic substrates by enhancing the expression of bone morphogenic protein 4 *via* NF–κB activity (Panizo et al., 2009). In addition, OPG deficiency enhanced the binding of RANKL to RANK, which exacerbated vascular calcification and osteoporosis in OPG–knockout mice (Bucay et al., 1998). While S100/calgranulins stimulate the production of RANKL, leading to increased osteoclast levels (Yoshida et al., 2009), the data from the study by Franke et al. demonstrated that AGEs increase the production of RAGE, RANKL, ALP, and osteocalcin mRNA in human osteoblasts obtained from cancellous bone (Franke et al., 2007). These findings indicate that the interaction of ligands with RAGE probably induces vascular calcification by activating the RANKL–RANK pathway by enhancing inflammatory cytokine levels *via* NF–κB activation. Thus, targeting RAGE seems to have the potential to inhibit vascular calcification and prevent cardiovascular events.

Ligand–RAGE interaction downregulates the production of prostacyclin (PGI2), an endogenous inhibitor of platelet aggregation, in endothelial cells and promotes *de novo* synthesis of PAI–1 to inhibit fibrinolytic activity and stabilize thrombi (Takenaka et al., 2006). Thus, RAGE activation is a potentially critical mechanism that enhances platelet aggregation and promotes the coagulation cascade, which is possibly linked to myocardial infarction and stroke in diabetic conditions. In addition, mechanical stimulation of the blood vessels during balloon dilation or surgical angioplasty induces inflammation, thrombus formation, smooth muscle proliferation and migration, and intimal thickening. Consequently, vascular restenosis, in–stent restenosis, and vascular allograft occlusion occur frequently. Ojima et al. found that balloon dilation of the internal carotid artery in rats caused marked intimal thickening and upregulation of RAGE expression, accompanied by AGE accumulation (Ojima et al., 2014). Therefore, these findings indicate that the engagement of RAGE with ligands triggers induction of vascular injury, inflammation, and thrombosis, thereby contributing to coronary artery disease and concomitantly inducing mechanical stimulation–induced intimal thickening. Thus, RAGE plays a pathological role in not only diabetes but also non-diabetic conditions.

## 10 RAGE–targeting latest therapeutic options

The development of therapeutic compounds aimed at inhibiting AGE formation has been ongoing for many years. Additionally, in recent years, there has been an increase in the research and development of RAGE–targeting therapies (Table 1). The soluble form of RAGE, sRAGE, sequesters circulating RAGE ligands to inhibit the engagement of membrane–bound RAGE and subsequent intracellular signal transduction. The administration of recombinant sRAGE has been shown to improve vascular and renal dysfunction (Wautier et al., 1996) and wound healing (Goova et al., 2001) in diabetic rodents. For clinical use in humans, a large amount of recombinant sRAGE is required; thus, using recombinant sRAGE in humans for the targeted therapy may be difficult to achieve from a cost standpoint. A RAGE–antagonist peptide (RAP) derived from HMGB–1 or S100A8 is also a candidate for RAGE–targeting therapy. RAP inhibits histological lung injury in an acute lung injury rodent model (Lee et al., 2018). RAP can bind to membrane–bound RAGE to competitively inhibit ligand binding to RAGE, and it can also be used as a drug delivery system to deliver the adiponectin gene as a therapeutic gene (RAP/APN) to RAGE–positive cells. Additionally, RAP/APN attenuates lung injury in a rodent model (Piao et al., 2020). Furthermore, small molecules targeting RAGE have been developed, and their efficacy has been confirmed in several rodent disease models. Manigrasso et al. screened a library of 58,000 small molecules and identified 13 small–molecule competitive inhibitors of the interaction between C–terminal RAGE (ctRAGE) and Dia–1. The compounds suppressed pro–inflammatory cytokine production and the activation of microphages in the LPS–induced inflammation model (Leerach et al., 2021) and N–CML–injected mice (Manigrasso et al., 2016). Additionally, these compounds preserved cardiac function in STZ–induced diabetic mice (Manigrasso et al., 2016).

**TABLE 1:** Characteristics of the latest RAGE-targeting therapeutic agents.

Agents	Mechanism of RAGE inhibition	Inhibitory effect on cell function	Disease model
Recombinant soluble RAGE	Trapping RAGE ligands	• Inhibiting the activation of MAPK, Rho GTPase, NF-κB, and AP-1	T2DM Panizo et al., (2009) and cardiac fibrosis Bucay et al., (1998)
		• Reducing ligands-induced inflammatory response, migration, and proliferation	
RAP	Preventing the ligands from binding to RAGE or using as a drug delivery system	• Suppressing NF-κB activation and pro-inflammatory cytokines (e.g., TNF-α, IL-1β, and IL-6) in LPS- or HMGB-1-treated macrophage	LPS-induced ALI Yoshida et al., (2009); Dwyer et al., (2012)
RAP /pAPN		• Attenuating acute lung injury	
Small molecule compound 13	Inhibiting the interaction between cytosolic tail of RAGE and Dia-1	• Inhibiting RAGE ligands- or PDGF-stimulated migration in smooth muscle cells	LPS-induced inflammation, T1DM Hirose et al., (2010), and the ligands-activated macrophage Xiong et al., (2011)
		• Reducing the production of TNF-α and IL-6 in response to the stimuli of ligands	
		• Inhibiting macrophage invasion by suppressing NF-κB and Rac-1	
Mutant RAGE peptide (S391A-RAGE362-404)	Inhibiting the formation of RAGE/AT1R complex	• Preventing angiotensin-II-induced RAGE activation	Atherosclerosis Weng et al., (2021)
		• Inhibiting angiotensin-II-stimulated production of ICAM-1, VCAM-1, TNF-α, and MCP-1	
AGEs-DNA-aptamer	Binding to AGEs, causing macrophage to phagocyte AGEs	• Suppressing pro-inflammatory cytokines (e.g., MCP-1 and TNF-α) and profibrotic cytokines (e.g., CTGF) with reduction in ROS levels	DM-N Linden et al., (2008) and vascular restenosis Brown et al., (2007)
RAGE-DNA-aptamer	Binding to RAGE and competitively inhibiting the engagement of RAGE with ligands	• Blocking the production of pro-inflammatory cytokines (MCP-1 and TNF-α), adhesion genes (ICAM-1 and VCAM-1), and profibrotic markers (Collagen I and CTGF)	DM-N Nakamura et al., (2009), HT-N Ando et al., (2013), and malignant melanoma Suga et al., (2011)
		• Reducing AGEs-induced ONOO^−^ production	
FPS-ZM1	Binding to V RAGE and blocking other ligands from binding	• Reducing MCP-1, TNF-α, IFN-β, and MMP-9 levels	DM-N Jeong et al., (2019), HT-N Zhao et al., (2021), AD Takenaka et al., (2006); Franke et al., (2007), and SARS-CoV-2 infection Hedrick et al., (2000)
		• Improving survival rate post SARS-CoV-2 infection	
		• Decreasing the amyloid tau protein deposition and mitigating inflammatory response	
RAGE vaccine	Producing RAGE-specific IgG antibody	• Improving glomerulosclerosis by reducing ICAM-1 and VCAM-1 levels	T1DM and T2DM Koga et al., (2021)

RAGE, receptor for advanced glycation endproducts; RAP, RAGE-antagonist peptide; AGE, advanced glycation endproducts; PDGF, platelet-derived growth factor.

As mentioned earlier, the activation of the angiotensin I receptor (AT1R) by Ang II triggers the transactivation of ctRAGE, activating NF–κB independent of the ligands binding to ectodomain RAGE. However, the mutant RAGE peptide S391A–RAGE362–404 inhibits RAGE transactivation to prevent Ang II–dependent inflammation and atherogenesis (Pickering et al., 2019). The RAGE–specific inhibitor FPS–ZM1 attenuated tubular injury with a reduction in the oxidative stress level and the production of TNF–α and IL–6 in STZ–induced diabetic rats (Sanajou et al., 2019). The upregulation of the expression of NADPH oxidase components, including Cyba, Nox1, Nox2, Nox4, and Ncf, was suppressed along with the suppression of IL–1β and TNF–α production in spontaneously hypertensive rats (Liu et al., 2020). Additionally, a novel option for RAGE inhibition is available, RAGE vaccination. Azegami et al. synthesized a RAGE partial peptide, produced by Eurofins Genomics, coupled to the keyhole limpet hemocyanin protein and identified that the IgG antibody specific for RAGE was produced after vaccinating rodents with the synthesized partial peptides. The antibody concentration remained high for 42 weeks. RAGE vaccination in db/db mice attenuated urinary albumin excretion, podocyte injury, and glomerular sclerosis, as well as reduced ICAM–1 and VCAM–1 production (Azegami et al., 2021).

**FIGURE 3:**
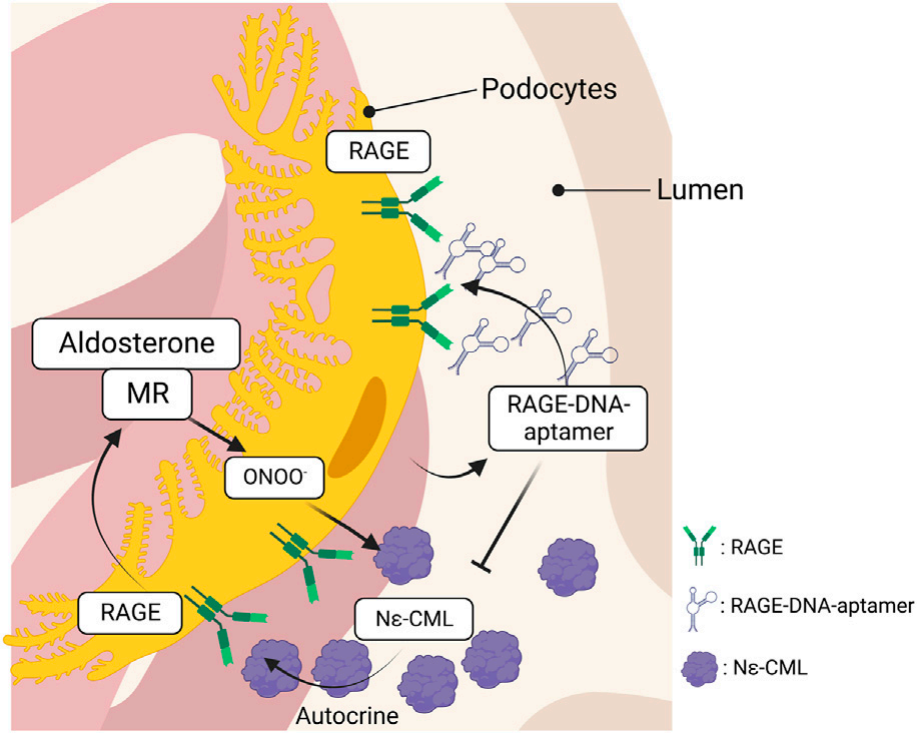
The interaction between RAGE and MR in podocytes. Aldosterone produces ONOO^−^ through the interaction with MR, which, in turn, promotes intracellular Nε-CML production in podocytes. RAGE is activated by the Nε-CML in an autocrine manner and the RAGE activation upregulates MR expression that forms a vicious cycle to induce podocytopathy. The vicious cycle is blocked by the inhibition of RAGE, leading to attenuate podocyte injury. RAGE, receptor for advanced glycation endproducts; MR, mineralocorticoid receptor.

Kaida et al. synthesized a DNA aptamer targeted against glyceraldehyde AGEs (AGE–aptamer) and identified glomerular sclerosis, renal AGE accumulation, and ROS production in STZ–induced diabetic mice, all of which were significantly attenuated by subcutaneous treatment with the AGE–aptamer (Kaida et al., 2013). Matsui et al. screened aptamers targeted against RAGE (RAGE–aptamer) and found that RAGE–DNA–aptamer inhibits urinary albumin excretion, glomerular sclerosis, and increase in the renal AGEs–RAGE level with a reduction in pro–inflammatory cytokine production (e.g., MCP–1, TNF–α, ICAM–1, and VCAM–1) (Matsui et al., 2017). The renin–angiotensin system is a hormonal cascade that regulates blood pressure, sodium retention, and tissue perfusion. Angiotensin stimulates the secretion and synthesis of aldosterone (Aldo) from the adrenal gland, which binds to the mineralocorticoid receptor (MR) to control sodium and water reabsorption. However, recent studies have demonstrated that renal MR activation is considerably linked to CKD progression (Artunc and Lang, 2014), suggesting that MR activation plays a central role in the progression of kidney injury. Taguchi et al. identified that Aldo induces intracellular production of Ne–CML *via* ONOO^−^production, which in turn, activates RAGE as an autocrine signal to form a vicious cycle in podocytes (Taguchi et al., 2018). However, the RAGE–DNA–aptamer blocked Aldo–induced RAGE activation by blocking the binding of N–CML to RAGE and preventing Aldo–induced podocyte injury (Figure 3). Furthermore, the RAGE–DNA–aptamer suppressed the proliferation and liver metastasis of malignant melanoma in nude mice (Nakamura et al., 2017). Thus, RAGE–DNA–aptamers can be a potent therapeutic option to inhibit RAGE signaling and can be used for preventing the progression of several diseases.

Glucocorticoids (GC) bind to glucocorticoid receptor (GR), a member of the nuclear receptor, which is expressed by all cell types. GC–GR signaling is known to suppress inflammatory response through multiple mechanisms (Nelson et al., 2003; Lim et al., 2014; Acharya et al., 2020). Interestingly, the treatment with GC suppresses RAGE–dependent NFkB activation in rodent model of acute lung injury (Hu et al., 2015), indicating that administration with GC might regulate RAGE–NFκB signaling and attenuate tissue damage and fibrosis. Besides, linagliptin, a dipeptidyl peptidase–4 (DPP–4) inhibitor, and empagliflozin, a sodium glucose transporter2 inhibitor, are widely used worldwide to control blood glucose level and prevent diabetic complications in patients with diabetes. They appear to have an inhibitory impact on AGEs–RAGE axis and protect organs in diabetic condition (Steven et al., 2017; Kaifu et al., 2018). Further, N–acetyl–seryl–aspartyl proline (AcSDKP) is known to have anti–fibrotic and anti–inflammatory effect in diabetic condition. AcSDKP is hydrolyzed by angiotensin converting enzyme (ACE); thus, serum concentration of AcSDKP is increased by treatment with ACE–I (Azizi et al., 1997). Not only does the combination therapy of ACE–I and AcSDKP strikingly suppresses renal fibrosis in diabetic CD–1 mice, but also oral administration with AcSDKP alone attenuates renal fibrosis *via* suppressing EndMT (Nagai et al., 2014; Nitta et al., 2016), suggesting that AcSDKP could become a potent therapeutic agent to prevent tissue fibrosis in diabetes. Downregulation of AcSDKP in diabetic mice is accompanied with the induction of renal DPP–4 (Srivastava et al., 2016), whereas linagliptin increases the concentration of renal AcSDKP independently of GLP–1 receptor in fibrotic kidneys (Hasan et al., 2019). Pharmacological inhibition of DPP–4 and the administration with AcSDKP modulate oxidative stress (Pejman et al., 2020), which is compatible with the previous study showing that RAGE–induced oxidative stress is regulated by linagliptin (Ishibashi et al., 2013). Those findings indicate that the administration with AcSDKP presumably regulates AGE–RAGE–induced intracellular oxidative stress, providing a protective effect in diabetic condition. However, whether AcSDKP has a direct impact on RAGE remains unknown; thus, future investigations regarding the relationship between AcSDKP and RAGE will be required.

## 11 Future directions and perspectives

AGE–targeted compounds to inhibit the production of AGEs are protective against diabetic complications. However, AGEs–targeted therapies have not been applied in the clinic due to adverse effects (Schalkwijk and Miyata, 2012). Meanwhile, with the advance of science and technology, RAGE–targeting therapeutic strategies other than sRAGE, have received recent attention. The current RAGE–targeting strategies against diabetic complications are promising, but further clinical studies are required to validate their efficacy and safety. Clinical trials with RAGE–targeting compounds such as TPP488 and ARO–RAGE are ongoing; thus, RAGE–targeting therapy is expected to be developed and investigated more in several clinical trials.

## 12 Conclusion

RAGE is a multiligand receptor belonging to the immunoglobulin superfamily. As RAGE is highly expressed in various cell types, there is increasing evidence that RAGE plays a pivotal role in the development of diabetes, diabetes–associated complications, kidney injury, inflammation, neurodegenerative disorders, and sepsis. With the development of RAGE–targeting agents, such as small molecules, aptamers, and RAGE antagonist peptides, in recent years, it has become possible to inhibit RAGE activity without any adverse effects. Our findings highlight the possibility of using a RAGE–targeted strategy for inhibiting diabetes–induced organ damage. As RAGE might be involved in inflammation post–SARS–CoV–2 infection (Jessop et al., 2022), the scope for RAGE–inhibition therapy for other diseases can be expanded. However, concerns regarding the long–term impact of RAGE blockade need to be addressed in future clinical studies. Further investigations are required for an improved understanding of which RAGE inhibitors are effective against diabetic complications in humans.
